# Elastic-Stiffness Coefficients of Titanium Diboride

**DOI:** 10.6028/jres.114.024

**Published:** 2009-12-01

**Authors:** Hassel Ledbetter, Takaho Tanaka

**Affiliations:** Mechanical Engineering University of Colorado Boulder, Colorado 80309; National Institute for Materials Science, Sengen, Namiki, Tsukuba, Ibaraki 305-0047 Japan

**Keywords:** Debye temperature, elastic constants, Grüneisen parameter, hexagonal crystal, monocrystal, sound velocities, titanium diboride, voids

## Abstract

Using resonance ultrasound spectroscopy, we measured the monocrystal elastic-stiffness coefficients, the Voigt *C*_ij_, of TiB_2_. With hexagonal symmetry, TiB_2_ exhibits five independent *C*_ij_: *C*_11_, *C*_33_, *C*_44_, *C*_12_, *C*_13_. Using Voigt-Reuss-Hill averaging, we converted these monocrystal values to quasiisotropic (polycrystal) elastic stiffnesses. Briefly, we comment on effects of voids. From the *C*_ij_, we calculated the Debye characteristic temperature, the Grüneisen parameter, and various sound velocities. Our study resolves the enormous differences between two previous reports of TiB_2_’s *C*_ij_.

## 1. Introduction

Currently, we see a renascence of research on transition-metal diborides. For example, well-known are many current studies on ReB_2_ and OsB_2_ as superhard materials, rivaling even diamond.

Titanium diboride’s physical and mechanical properties received review elsewhere [1, 2]. This compound is known well for low mass density, high hardness, high melting point, low electrical resistivity, good thermal conductivity, and good chemical inertness. However, the problems in preparing full-dense, high-quality monocrystals preclude their extensive study. Akimitsu and colleagues [3] reported superconductivity at 40 K in a same-crystal-structure companion compound: MgB_2_.

The importance of elastic-stiffness coefficients for both science and technology also received review [4].

Titanium diboride’s monocrystal elastic stiffnesses appeared in two reports. The values of Gilman and Roberts [5] depart strongly from the more recent report of Spoor and colleagues [6], mainly in the off-diagonal *C*_12_ and *C*_13_. The first measurements were made by a pulse-echo method, the second by resonance ultrasonic spectroscopy, but by a very different mechanical setup than used in the present study.

Here, we report a third set of measurements, which agree closely with the Spoor et al. results. From our *C*_i j_ measurements, we estimate the Debye characteristic temperature, the quintessential harmonic-lattice property. Also, we estimate the Grüneisen parameter, the quintessential anharmonic property.

**Fig. 1:**
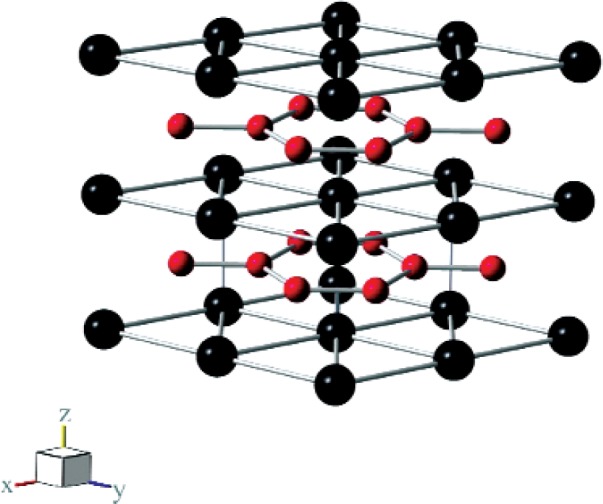
Schematic crystal structure of titanium diboride (AlB_2_ type, C32, hexagonal, P6/mmm, hP3, M = 1, D 16h, No. 191). Small spheres represent boron atoms, large spheres titanium atoms. The hexagonal boron net resembles strongly that of graphitic carbon; thus we expect strong interatomic bonding within the boron net. The titanium atoms nest in interstices provided by the boron net. The axial ratio equals 3.228/3.028 = 1.066, relatively high for the AlB_2_-compound group. Each Ti atom is surrounded by twelve equidistant boron atoms. Each boron atom has three boron atoms at a short distance, and six titanium atoms at a much longer distance.

Figure 1 shows the TiB_2_ crystal structure, obviously a layered structure suggesting strong elastic anisotropy, not observed as shown below, creating a conundrum.

## 2. Measurements

### 2.1 Crystal

An oriented parallelepiped specimen 1.8 mm × 2.3 mm × 3.9 mm was prepared from a larger crystal grown by a floating zone method [7, 8]. Fluorescent x-ray analysis revealed no significant impurities. The exact stoichiometry was TiB_1.97_. The crystal faces with respect to the above dimensions were [2 – 1 – 10], [−1100], [0001]. These directions represent an *a*-axis, the *c*-axis, and the direction orthogonal to both. Laue x-ray diffraction confirmed these orientations within 1°.

### 2.2 Method

**Fig. 2:**
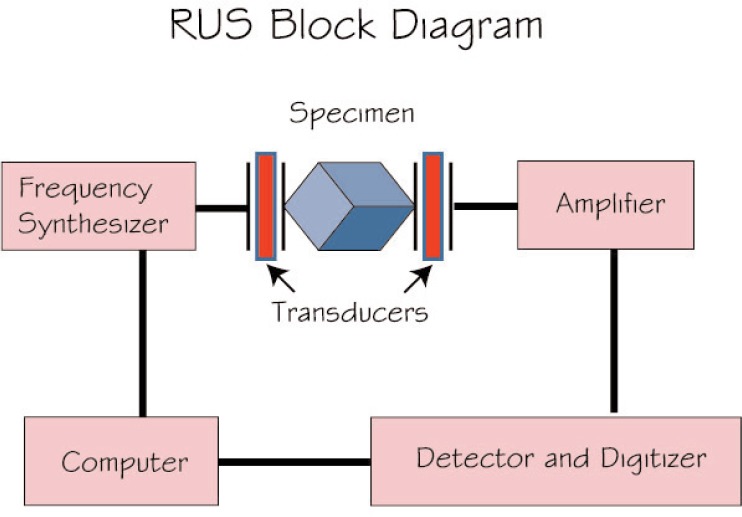
Schematic measurement setup. Specimen (parallelepiped) is clamped loosely between two piezoelectric transducers. One transducer is swept through frequency. The second transducer detects macroscopic resonance frequencies, which depend on specimen shape, size, mass, and elastic-stiffness coefficients, the *C*_ij_. Courtesy of A. Migliori (Los Alamos National Laboratory).

**Fig. 3:**
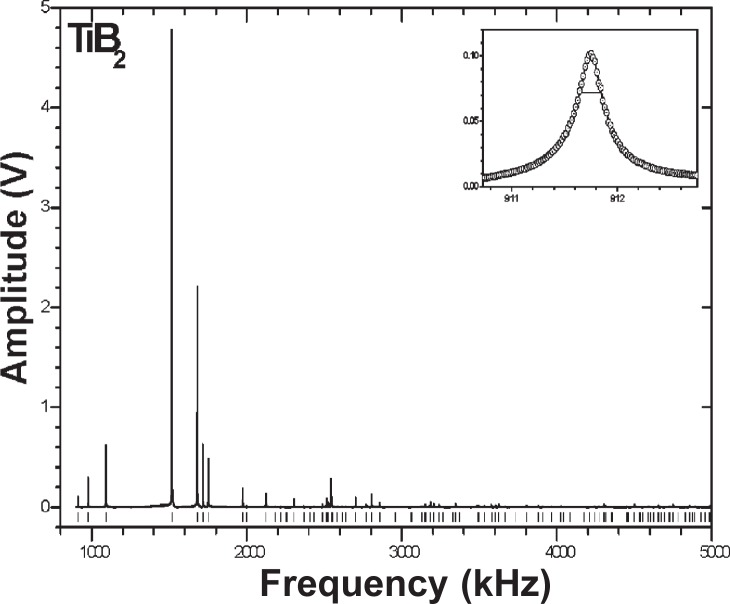
Macroscopic resonance spectrum. Resonance frequnecies *f*_n_ yield *C*_ij_ by an inverse-problem calculation. Highly overdetermined, the problem uses about one hundred resonance frequencies *f*_n_ to determine the five independent *C*_ij_. Vertical bars at bottom show predicted resonance frequencies. The inset shows a resonance-peak profile, with a Lorentzian shape, the half-power width giving the internal friction *Q* ^−1^ i j, the imaginary part of the total *C*_ij_.

To measure the *C*_ij_, we used resonance ultrasound spectroscopy, summarized in Fig. 2 [9–11]. Briefly, one clamps lightly a regular-shape (cube, cylinder, cube, parallelepiped) specimen between two piezoelectric transducers. One transducer is swept through frequency and the second transducer detects the specimen’s macroscopic vibration frequencies (Fig. 3). Frequencies of a specimen are determined by five factors: (1) shape, (2) size, (3) mass or mass density, (4) elastic-stiffness coefficients *C*_ij_, and (5) crystal-axis orientation relative to macroscopic shape. Thus, by measuring the resonance frequencies *f*_n_, one can determine by an inverse calculation the stiffnesses *C*_ij_. The problem is strongly overdetermined: about one hundred *f*_n_ to determine five *C*_ij_. Not reported here, one can also determine the complete internal-friction tensor $Q_{ij}^{-1}=\Delta f_{n}/f_{n}=C_{ij}^{*}/C_{ij}$, where Δ*f*_n_ denotes resonance-peak width, $C_{ij}^{*}$ the imaginary part, and *C*_ij_ the real part of the total ${\tilde{C}}_{ij}$ tensor. Well-described elsewhere [12], the inverse problem involves Lagrangean minimization, the Rayleigh-Ritz method, and a least-squares procedure for measured and deduced *f*_n_ values.

We determined mass density by careful mass and size measurements: *ρ* = 4.502 ± 0.016 g/cm^3^. This compares with reported x-ray mass densities of 4.504 to 4.53. We ascribe the differences to heavier impurities on the Ti sites. From this mass density, we concluded our specimen contains no significant void content. From the sharp resonance peaks (Fig. 3), we concluded our specimen contains few cracks.

### 2.3 Errors

Errors arise from many sources: crystal orientation, crystal dimensions, nonparallelism, the inverse problem (measured-frequency to *C*_ijkl_ conversion), specimen-transducer interactions. Several authors described these errors elsewhere, especially the first reference [13–15]. The effect of the slight (1 %) departure from stoichiometry is hard to estimate. Because of the strong covalent bonding within the boron planes, we conjecture a very small error arising from an occasional missing boron atom.

## 3. Results and Discussion

Table 1 shows our principal results: the *C*_ij_ and their uncertainties. (Because the two previous reports omitted error estimates, very detailed comparisons are precluded.) Only five *C*_ij_ are independent because *C*_66_ = (*C*_11_ −*C*_12_)/2. As usual (they fail to correspond directly to a phonon), the off-diagonal *C*_ij_ (*C*_12_ and *C*_13_) show the largest uncertainties. Table 1 also gives the Spoor et al. results [6], which differ from ours by an average of 2.2 %. The average uncertainty in our six *C*_ij_ is 0.7 %. The close agreement between our results and the Spoor et al. results suggests strongly that the earlier Gilman–Roberts results [5] are wrong, the largest discrepancies occurring in the off-diagonal *C*_ij_: *C*_12_ and *C*_13_. Further support for the correctness of the Ledbetter-Tanaka/Spoor et al. results arises from *ab initio* calculations that yielded *B* = 251 GPa and *ν* = 0.12 [16], versus 417 and 0.32 for Gilman-Roberts.

Table 1 also shows the principal Young moduli *E*_ii_ computed by

$$E_{ij}=S_{ij}^{-1}.$$ (1)

Here *S*_ij_ denotes the elastic-compliance tensor, the tensor inverse of the *C*_ij_. As we expect from *C*_11_ > *C*_33_, *E*_11_ exceeds *E*_33_ ; that is, TiB_2_ is much stiffer within the basal plane than along the *c*-axis. Obviously, this relates to the crystal structure where the covalent-bonded boron atoms lie in the plane perpendicular to *x*_3_.

Table 1 shows also the three principal Poisson ratios *ν*_ij_ computed by

$$\nu_{ij}=-S_{ij}/S_{\mathrm{ii}}.$$ (2)

Within the boron plane, the Poisson ratio *ν*_12_ is extremely low, reflecting strong covalent bonding and the strong resistance of boron atoms to change their bond angles. The *ν*_13_ Poisson ratio is only slightly below normal, indicating weaker bonds out of the boron-atom planes than those within the planes.

The shear elastic anisotropy of hexagonal crystals can be expressed in various ways. The simplest is *C*_66_/*C*_44_, 1.15 for TiB_2_, thus weak elastic anisotropy (the isotropic case corresponding to 1.00). Because the Young modulus depends so strongly on the shear modulus, a Young-modulus variation with direction also serves as an effective shear-anisotropy indicator. Spoor and colleagues showed a Young-modulus representation surface; it is nearly spherical [6]. From the alternating-layer boron-titanium crystal structure (Fig. 1), one expects higher elastic anisotropy than one finds.

The lower part of Table 1 gives the averaged-over-direction quasiisotropic elastic constants obtained from the *C*_ij_ by a Voigt-Reuss-Hill average [17]. These constants include longitudinal modulus *C*_L_, bulk modulus *B*, shear modulus *G*, Young modulus *E*, and Poisson ratio *ν*. These are the elastic constants appropriate to a full-density nontextured polycrystalline aggregate in which the grain boundaries cause no softening. Grain size produces no effect on elastic constants if it is small relative to specimen size. (A reviewer pointed out that nanosize grains may soften the elastic constants.) The most unusual feature of our results is the high *G*/*B* ratio, thus low Poisson ratio. Our nonpublished results on a YB_66_ monocrystal gave an averaged-over-direction (Kröner method) Poisson ratio of 0.13. For boron, a handbook value is *ν* = 0.089 [18]. Both the bulk and shear moduli of TiB_2_ exceed considerably (by 30 % to 40 %) the handbook values of boron: 248/179 = 1.39 and 264/203 = 1.30, showing the strong interatomic bonds in TiB_2_, both for extension-compression and for shear-bending. Beside implying a low Poisson ratio, the very high *G*/*B* ratio holds implications for several other crystal-bonding properties: covalency, Cauchy-relation departure, many-body forces, and others. We plan to discuss all these elsewhere.

The elastic stiffnesses yield three useful sound velocities: the longitudinal velocity,

$$\nu_{1}={\left(C_{L}/\rho \right)}^{1/2}={\left[\frac{B+\left(4/3\right)G}{\rho }\right]}^{1/2},$$ (3)

the shear or transverse velocity

$$\nu_{s}={\left(\frac{G}{\rho }\right)}^{1/2},$$ (4)

and the mean velocity (as defined by Debye)

$$\frac{3}{\nu_{m}^{3}}=\frac{2}{\nu_{t}^{3}}+\frac{1}{\nu_{1}^{3}},$$ (5)

We found *ν*_l_ = 1.54, *ν*_s_ = 0.766, and *ν*_m_ = 0.835 cm/μs.

From the *C*_ij_ and the mass density, we can compute the acoustic Debye characterisitic temperature *Θ*_D_. At zero temperature, the acoustic *Θ*_D_ becomes identical with the calorimetric *Θ*_D_ [19]. *Θ*_D_ is proportional to the mean sound velocity:

$$\Theta_{D}=K\nu_{m},$$ (6)

where *ν*_m_ denotes mean sound velocity and K is given by

$$K=\frac{h}{k}{\left(\frac{3}{4\pi V_{a}}\right)}^{1/3}.$$ (7)

Here *h* denotes Planck’s constant, *k* Boltzmann’s constant, and *V*_a_ atomic volume. The velocity *ν*_m_ comes from the integration over all directions:

$$3\nu_{m}^{-3}=\int \sum_{\alpha =1,3}\nu_{\alpha }^{-3}d\Omega /4\pi .$$ (8)

Here *ν*_1_ denotes the quasilongitudinal wave velocity, *ν*_2_ and *ν*_3_ the quasitransverse wave velocities, and *d*Ω an increment of solid angle. Equation (8) can not be integrated analytically, and numerous numerical and approximate methods have been used for its solution. Phase velocities *ν_a_* are roots of the Christoffel equations:

$$\begin{array}{l} \det\left(C_{ijkl}x_{j}x_{k}-\rho \nu^{2}\delta_{il}\right)=0.\\ \left(\text{sum over repreated indices}\right) \end{array}$$ (9)

This expression follows from equations of motion for plane, monochromatic waves, where *ρ* denotes mass density, *C*_ijkl_ fourth-rank elastic-stiffness tensor, *x*_i_ components of the unit wave vector relative to the cubic axes, and *δ*_il_ the Kronecker operator. Equation (9) usually yields three distinct real roots *ρν* ^2^.

We computed an exact *Θ*_D_ by using a distribution of 489 vectors proposed by Overton and Schuch [20], which we distributed over the usual 48 [100]–[110]–[111] stereographic unit triangles, thus a total of 23 472 directions. We obtained *Θ*_D_ = 1217 K. This result differs enormously from the handbook calorimetric value, 1576 K. Often, calorimetric values contain large errors because of large extrapolations to zero temperature and/or large uncertainties in the lowest-temperature lattice specific heat.

From *B* and from handbook values of heat capacity *C*, volume thermal expansivity *β*, and volume *V*, we computed the effective Grüneisen parameter:

$$\gamma =B_{S}\beta V/C_{P}.$$ (10)

We obtained *γ* = 1.71, the handbook values for boron and titanium being 1.85 and 1.33. Alternative gammas can be computed when one knows the third-order elastic-stiffness coefficients, the *C*_ijklmn_ [21]. The few gammas known for compounds similar to TiB_2_ preclude any comparisons. Following Pearson’s reasoning [22], the Ti-B bonds would lead to *γ* = 2.

Finally, because some authors suspect voids/cracks in TiB_2_, we want to describe briefly how the above full-dense quasiisotropic elastic constants would change with voids. Focusing on Al_2_O_3_, Ledbetter, Lei, and Datta [23] gave a theory for void effects on elastic constants. Principal results include the following: Voids soften the bulk modulus more than the shear modulus. In the dilute limit, for spherical voids, our results agree with the classical results of Mackenzie [24]:

$$\frac{\Delta B}{B}=-3c,\;\left(\nu_{0}=1/3\right)$$ (11)

$$\frac{\Delta G}{G}=-\frac{15}{8}c.\;\left(\nu_{0}=1/3\right)$$ (12)

Here, *c* denotes void volume fraction and *ν*_0_ the void-free-state Poisson ratio. However, we emphasize that the often-used rule-of-thumb that elastic stiffness varies as mass density is true only in the dilute limit. Void shape plays a key role, especially if the voids possess an oblate-spheroid (disc) shape. Dunn and Ledbetter [25] focused on the interesting, unexpected effects of voids and cracks on the Poisson ratio. Other authors addressed this problem using various approximations [26].

## 4. Conclusions

1. Our elastic-stiffness-coefficient measurements on titanium diboride support the report of Spoor and colleagues rather than the older (now handbook) values of Gilman and Roberts. The principal differences in the two previous *C*_ij_ sets lie in *C*_12_ and *C*_13_.
2. From the *C*_ij_ we computed several additional useful physical properties:
  - sound velocities;
  - Debye temperature;
  - Grüneisen parameter.
3. Our computed acoustic Debye temperature, 1217 K, is about 20 % lower than the handbook calorimetric value.
4. Our computed Grüneisen parameter, 1.71, suggests the importance of Ti-B bonds along with B-B bonds.
5. Using the *C*_66_/*C*_44_ ratio as a shear-mode elastic-anisotropy criterion, TiB_2_ shows low shear-mode anisotropy, 1.15. A surprise because of the B-Ti-B … layer crystal structure.
6. Our elastic constants estimated for a full-dense polycrystal depart strongly from those given in Munro’s review [2], especially in the uncertainties. For example, Munro proposed a 70 % uncertainty in the Poisson ratio and a 24 % uncertainty in the bulk modulus. Our polycrystal results differ from the Spoor et al. result by only 2 %. This finding agrees with the well-known fact that modern measurement methods give the elastic-stiffness coefficients easily within one percent.
7. Finally, we are surprised by the low elastic shear anisotropy shown by such a strongly layered crystal structure. Reference [8] gives a possible explanation. Part of the boron-atom *p*-obitals lie in a *p-d* hybridized band, weakening the *p*-electron contribution to B-B bonding. If the bonding weakens, the anisotropy decreases.

## Figures and Tables

**Table 1:** Monocrystal and polycrystal elastic constants of titanium diboride. Unless specified, all units are GPa, except for the Poisson ratios, *ν*, which are dimensionless

Present		Gilman - Roberts	Spoor et al.^b^
*ρ* (g/cm^3^)	4.502 ± 0.016		
Monocrystal elastic constants			
*C*_11_	654.4 ± 1.9	690	660
*C*_33_	458.1 ± 1.4	440	432
*C*_44_	262.6 ± 0.3	250	260
*C*_66_	302.7 ± 0.7	140	306
*C*_12_	48.98 ± 1.4	410	48
*C*_13_	95.25 ± 0.55	320	93
*E*_11_	633.3 ± 2.2	389	639
*E*_33_	432.3 ± 1.7	254	408
*ν*_12_	0.0460 ± 0.0022	0.3877	0.0437
*ν*_13_	0.1984 ± 0.0015	0.4453	0.2059
*ν*_31_	0.1354 ± 0.0010	0.2909	0.1314
Debye characteristic temperature			
*Θ*_D_ (K)	1217 ± 6	989	1211

Voigt-Reuss-Hill-average quasiisotropic (polycrystal) elastic constants			
*C*_L_	599.9	642	593
*B*	247.5	417	244
*G*	264.3	169	262
*E*	584.7	446	579
*ν*	0.1063	0.3219	0.1037

a Ref. 5.

b Ref. 6.
